# Trypanosomatid RACK1 Orthologs Show Functional Differences Associated with Translation Despite Similar Roles in *Leishmania* Pathogenesis

**DOI:** 10.1371/journal.pone.0020710

**Published:** 2011-06-03

**Authors:** Kohelia Choudhury, Daviel Cardenas, Ashok K. Pullikuth, Andrew D. Catling, Ashok Aiyar, Ben L. Kelly

**Affiliations:** 1 Department of Microbiology Immunology and Parasitology, Louisiana State University Health Sciences Center, New Orleans, Louisiana, United States of America; 2 Department of Pharmacology and Experimental Therapeutics, Louisiana State University Health Sciences Center, New Orleans, Louisiana, United States of America; 3 Stanley S. Scott Cancer Center, Louisiana State University Health Sciences Center, New Orleans, Louisiana, United States of America; University of Georgia, United States of America

## Abstract

RACK1 proteins belong to the eukaryote WD40-repeat protein family and function as spatial regulators of multiple cellular events, including signaling pathways, the cell cycle and translation. For this latter role, structural and genetic studies indicate that RACK1 associates with the ribosome through two conserved positively charged amino acids in its first WD40 domain. Unlike RACK1s, including *Trypanosoma brucei* RACK1 (TbRACK1), only one of these two positively-charged residues is conserved in the first WD40 domain of the *Leishmania major* RACK1 ortholog, LACK. We compared virulence-attenuated *LACK* single copy (*LACK/-*) *L. major*, with *L. major* expressing either two *LACK* copies (*LACK/LACK*), or one copy each of *LACK* and *TbRACK1* (*LACK/TbRACK1*), to evaluate the function of these structurally distinct RACK1 orthologs with respect to translation, viability at host temperatures and pathogenesis. Our results indicate that although the ribosome-binding residues are not fully conserved in LACK, both LACK and TbRACK1 co-sedimented with monosomes and polysomes in *LACK/LACK* and *LACK/TbRACK1 L. major*, respectively. *LACK/LACK* and *LACK/TbRACK1* strains differed in their sensitivity to translation inhibitors implying that minor sequence differences between the RACK1 proteins can alter their functional properties. While biochemically distinguishable, both *LACK/LACK* and *LACK/TbRACK1* lines were more tolerant of elevated temperatures, resistant to translation inhibitors, and displayed robust pathogenesis *in vivo*, contrasting to *LACK/-* parasites.

## Introduction

Eukaryote RACK1 proteins are highly conserved members of the WD40-repeat protein family adopting a modular seven-bladed ß-sheet propeller structure [1], [2] that regulate a variety of cellular pathways [3], [4], [5]. Studies in mammals, yeasts, plants and the trypanosomatid protozoan, *T. brucei*, confirm important functions for RACK1 proteins in cell signaling, division, differentiation and translation [6], [7]. Studies of the RACK1 orthologs CPC2 and ASC1 in *Schizosaccharomyces pombe* and *Saccharomyces cerevisiae*, respectively, indicate these proteins associate with the translation machinery and regulate protein expression [7], [8].

The physiological importance of RACK1 proteins is well documented. *CPC2*-deficient *S. pombe* display a delayed progression through the cell cycle, and an aberrant response to environmental stimuli that induce G1 arrest [9]. These findings, together with data suggesting that ASC1 is important for adhesin-dependent growth and temperature tolerance in *S. cerevisiae*, demonstrate the importance of RACK1 proteins in cellular responses to environmental changes [10], [11]. Recent structural studies have identified a ribosome-binding motif consisting of a tripeptide arginine, aspartate, and lysine (RDK) in the WD40 domain of the *S. cerevisiae* RACK1 protein [12], [13]. This motif is highly conserved in RACK1 orthologs of other eukaryotes, including *T. brucei* RACK1 (TbRACK1). Paradoxically, although TbRACK1 is evolutionarily closely-related to the *Leishmania* RACK1 ortholog, LACK, the RDK tripeptide is not conserved.

Mutation of the conserved RDK in yeast RACK1 decreases tolerance to translation inhibitors, confirming the functional importance of ribosomal association with RACK1 via this motif [12]. Studies in mammalian cells demonstrate RACK1 associates with both ribosomes and protein kinase C (PKC) thus linking cell signaling cascades to translation [14], [15]. Further, RACK1 is also required for PKC-dependent phosphorylation and release of mammalian translation initiation factor 6 (eIF6) from the 60S ribosomal subunit, prior to assembly of the 80S ribosome [16], [17]. Studies in other eukaryotes also link RACK1 functions in translation to other cellular pathways. For example, data suggest that the function of TbRACK1 in cytokinesis is dependent upon its function in translation [18], [19]. The same studies also identified eukaryote elongation factor 1A (eEF1A) as a TbRACK1-binding protein; consistent with TbRACK1 functioning in translation elongation, *T. brucei* depleted for TbRACK1 display an increased sensitivity to the elongation inhibitor, anisomycin [18].

Our previous studies demonstrate that the *Leishmania major* RACK1 ortholog, LACK, is essential for parasite viability, survival at host temperatures, and robust infection of host macrophages [20]. The diploid genome of *L. major* has four copies of the *LACK* gene, organized as two tandem copies arranged head-to-tail on each homologous chromosome. These gene copies are indistinguishable in stage-specific expression, and are predicted to express proteins of identical sequence [20]. One allele, containing two of these four copies can be deleted without affecting viability or pathogenesis relative to wild-type (WT) *L. major* [20]. Targeted deletion of a third copy of *LACK* results in parasites with reduced levels of LACK that show reduced viability and severely attenuated virulence [20]. Parasites with three *LACK* copies deleted are referred to as *LACK/-* in this report. In contrast, targeted replacement of a third *LACK* copy with an *Xho* I restriction site-tagged *LACK* gene yields fully viable parasites [20]. These *L. major* lines, referred to as *LACK/LACK* in this report, contain one endogenous *LACK* copy, followed by a second, targeted *LACK* copy downstream, thus maintaining LACK expression from two *LACK* gene copies. Multiple attempts to delete all four copies of *LACK* failed, indicating that at least one copy of LACK is essential for parasite survival. Despite its importance in parasite viability and virulence, molecular mechanisms underlying LACK function in *Leishmania* have not yet been elucidated.

Although eukaryotic RACK1 orthologs are highly conserved, recent studies have identified subtle species-specific functional motifs [21], [22]. Some of these species-specific functional differences may result from sequence divergence. For example, although an RDK ribosome-binding motif is conserved in yeast, mammalian and *T. brucei* RACK1 orthologs, it is not conserved in LACK proteins *of Leishmania sp.*; in the latter, the highly conserved lysine is substituted with a glycine. Therefore, we hypothesized that LACK's ribosome association may differ from that observed for RACK1 in other eukaryotes, with implications for its function in translation.

We reasoned that the altered RDK motif in LACK might be compensated by other sequence alterations in LACK that maintain its structure and function. Therefore, rather than mutate the putative ribosome-binding motif in LACK, we replaced the second copy of *LACK* in *LACK/LACK* parasites with *TbRACK1*, to create a line referred to as *LACK/TbRACK1* in this report. By doing so, any differences observed between LACK and TbRACK1 could be attributed to differences in function rather than effects of mutation-induced disruption of protein structure. We evaluated the viability and virulence of *LACK/TbRACK1* parasites in comparison to *LACK/LACK* parasites, and identified differences in LACK's and TbRACK1's protein synthetic functions using translation inhibitors, and polysome association.

## Results

### LACK-deficient *L. major* show cell cycle defects at mammalian temperatures

**Figure 1:**
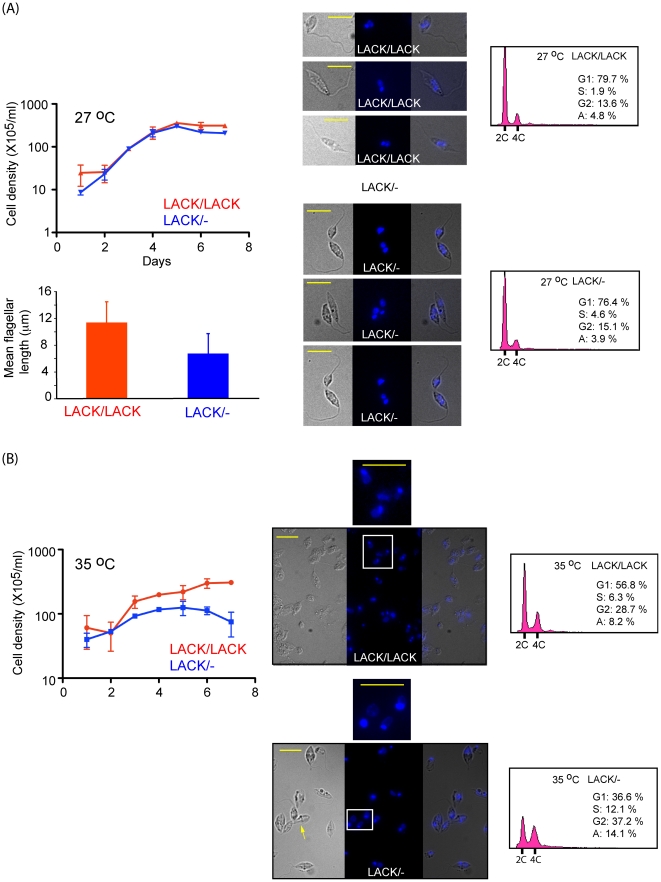
The effect of host temperature upon *LACK*-deficient *L. major* growth, morphology and the cell cycle. Parasites were seeded in medium 199/10% FBS at 5×10^5^/ml or 1×10^6^/ml and incubated at either 27°C (A) or 35°C (B), respectively. Cell densities were determined daily for seven days by enumeration of diluted parasites using a hemocytometer and plotted as indicated. At day 4 of incubation, culture samples were removed, diluted, and the parasites examined microscopically at 630× magnification for cellular morphology and DNA content by differential interference contrast microscopy (DIC) (left panels) and fluorescence microscopy (middle panel). A merged DIC/fluorescence image is shown in the right panel. Flagella lengths were determined using ImageJ software and indicated by the bar-chart in (A), using sample sizes of 13 and 9 for *LACK/LACK* and *LACK/-* promastigotes, respectively. White boxes in (B) indicate areas of images enlarged for clarity, as shown at right of lower panels. Scale bars (10 µm) are denoted by short yellow lines. Yellow arrow indicates rounded, enlarged morphologies of *LACK/-* parasites as described in the text. The cell cycle of day 4 parasites was also analyzed by flow cytometry, gating out cell doublets, at 27°C and 35°C, as shown in the right panels. % of the cell population corresponding to cell cycle phases are indicated: G1 (Gap_1_), S (Synthesis), G2 (Gap_2_). Aneuploid cells (A) with a DNA content greater than 4C are also indicated (A).

Previously, we demonstrated that *LACK/- L. major* lines caused dramatically attenuated disease in infected mice, and were significantly less viable at mammalian body temperature *in vitro* [20]. In contrast, *LACK/LACK* lines were as virulent as WT *L. major* in infected mice, and displayed no defect in viability at mammalian body temperature [20]. To further investigate these defects, we compared the morphology and DNA content of *LACK/LACK* and virulence-attenuated *LACK/- L. major* isolated at day 4 from promastigote cultures grown at 27°C and 35°C, using immunofluorescence microscopy. These results, shown in Figure 1, indicate that *LACK/-* parasites display an altered morphology relative to *LACK/LACK* parasites at both temperatures. At 27°C (Figure 1A), *LACK/-* parasites appear rounded with shorter flagellae; the latter phenotype is indicated by the bar-graph in Figure 1A. At 35°C (Figure 1B), *LACK/-* parasites were enlarged relative to *LACK/LACK* parasites (yellow arrow), with apparently greater nuclear and kinetoplast DNA content as evaluated by DAPI staining. This altered morphology coincided with a growth defect at 27°C, which was greatly accentuated at 35°C.

To further examine and quantify the difference in DNA content, we compared *LACK/LACK* and *LACK/-* lines by flow cytometric analyses of log-phase cells stained with propidium iodide (Figure 1, right panels as indicated). As shown in the figure, the cell-cycle profiles of both *LACK/LACK* and *LACK/-* parasite lines were comparable at 27°C, with a 2C∶4C ratio of approximately 6∶1. In contrast, significant differences were observed at 35°C. At this temperature *LACK/LACK* parasites displayed a 2C∶4C ratio of 2∶1, consistent with an accumulation in G2 and M as observed by others [19]. This accumulation was strikingly pronounced in *LACK/-* parasites, resulting in a 2C∶4C ratio of 1∶1. We interpret this difference to indicate that *LACK/-* parasites have an impaired stress response relative to *LACK/LACK* parasites. Coincidentally, TbRACK1-depleted *T. brucei* also accumulate post-S phase, and are blocked in cytokinesis [19]. Based on these empirical observations, LACK and TbRACK1 may have equivalent functions in the cell-cycle of these related trypanosomatids. Because the cell-cycle functions of TbRACK1 are dependent on its role in translation, we tested whether LACK co-sedimented with monosomes and polysomes as observed for other RACK1 orthologs.

### LACK co-sediments with a ribosomal protein in Leishmania monosomes and polysomes

**Figure 2:**
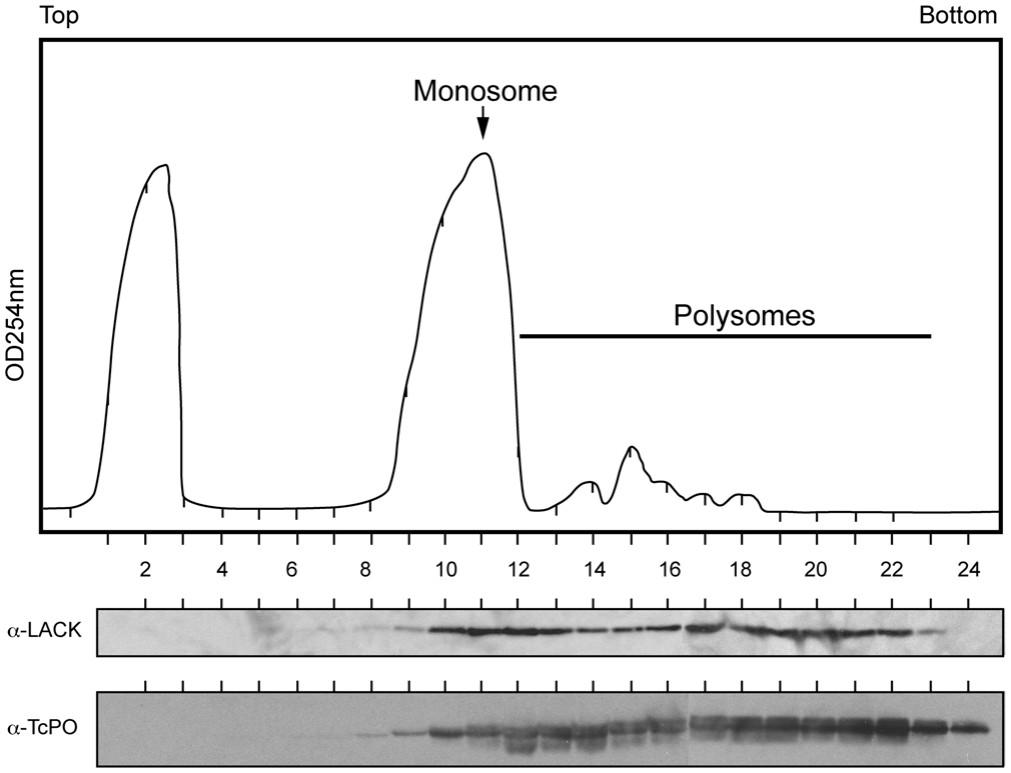
Sucrose gradient polysome analysis of LACK. *L. major* lysates were prepared for sucrose gradient fractionation as described in Materials and Methods, then applied to a 10–50% sucrose step gradient and centrifuged for 160 min at 35,000 rpm. Following rRNA monitoring (OD_254_) and fractionation of the centrifuged gradient into 0.5 ml aliquots, proteins were purified from each fraction by methanol/chloroform precipitation, resolved on SDS/PAGE gels, blotted and probed with either anti-LACK antisera or anti-TcPO antisera. Fractions are numbered relative to the top and bottom of the sucrose gradient, as indicated.

The RDK ribosome-binding motif is conserved in other RACK1 proteins, but is altered in LACK. Therefore, we tested whether LACK associates with *Leishmania* ribosomes by co-sedimentation analyses on discontinuous sucrose gradients (Figure 2). As shown in the figure, LACK co-sedimented in monosome and polysome fractions with the 60S ribosomal stalk protein PO [18], but was absent from low molecular weight fractions at the top of the sucrose gradient. Although we clearly observe a monosome peak, and polysome-containing fractions, we did not observe strongly distinct 40S and 60S ribosome subunit peaks, similar to previous findings in *L. infantum* [23]. In control experiments (data not shown), treatment with EDTA disrupted this profile, and resulted in LACK redistributing to the top of the gradient. It is of interest to note that the distribution of LACK in these gradients differs from the distribution of other RACK1 proteins, such as those from *S. pombe* and *T. brucei*. For example, TbRACK1, the LACK ortholog from *T. brucei*, a trypanosomatid closely related to *Leishmania*, can also be detected in non-ribosomal fractions. LACK's distribution closely resembles that observed for the *S. cerevisiae* RACK1 ortholog, which only associates with ribosome-containing fractions.

These data indicate that although the RDK motif is not conserved in LACK, it continues to associate with the *Leishmania* protein synthesis machinery. Three possibilities could account for this association: 1) LACK associates with ribosomes in a manner distinct from the other RACK1 proteins; 2) LACK contains other sequence changes that compensate for the absence of the RDK motif; or 3) there are compensatory changes in other components of the *Leishmania* translation apparatus that permit association with LACK. One way to dissect these possibilities is by replacing LACK with another RACK1 protein known to be functional in its native context.

If other components of the *Leishmania* translation apparatus have compensatory changes to permit association with LACK, it is expected that these changes would prevent this heterologous RACK1 protein from functioning in the context of *Leishmania*. If LACK contains compensatory mutations elsewhere within it to permit association with the same ribosomal site as the other RACK1 proteins, then too it is predicted that the heterologous RACK1 protein will not function in *Leishmania*. However, if LACK and the heterologous RACK1 protein functionally associate with ribosomes at distinct sites or by distinct mechanisms, it is anticipated that the heterologous RACK1 protein will substitute for LACK in *Leishmania*. An implication of the latter possibility is that inhibitors of the protein synthesis machinery may have differential effects on LACK and the heterologous RACK1 protein.

*Leishmania* require at least one copy of *LACK* to survive, and *LACK/LACK* parasites cannot be distinguished in viability and virulence from WT *L. major*. Therefore, we replaced one copy of *LACK* in *LACK/LACK* parasites with the RACK1 protein from *T. brucei*, TbRACK1. We chose TbRACK1 because *Leishmania* and *T. brucei* are closely related trypanosomatids; yet, the potential ribosome-binding motif is conserved in TbRACK1, but not LACK. Elegant studies by Ruben and co-workers have characterized the function of TbRACK1 in depth [18], further increasing the value of comparing these two proteins from closely related parasites. We were also curious to determine whether TbRACK1 would distribute to non-ribosomal fractions in *Leishmania*, as it does in *T. brucei*. A failure to do so would indicate that the intracellular milieu within these trypanosomatids differs.

### Targeted replacement of a LACK gene copy with TbRACK1 in *L. major*

**Figure 3:**
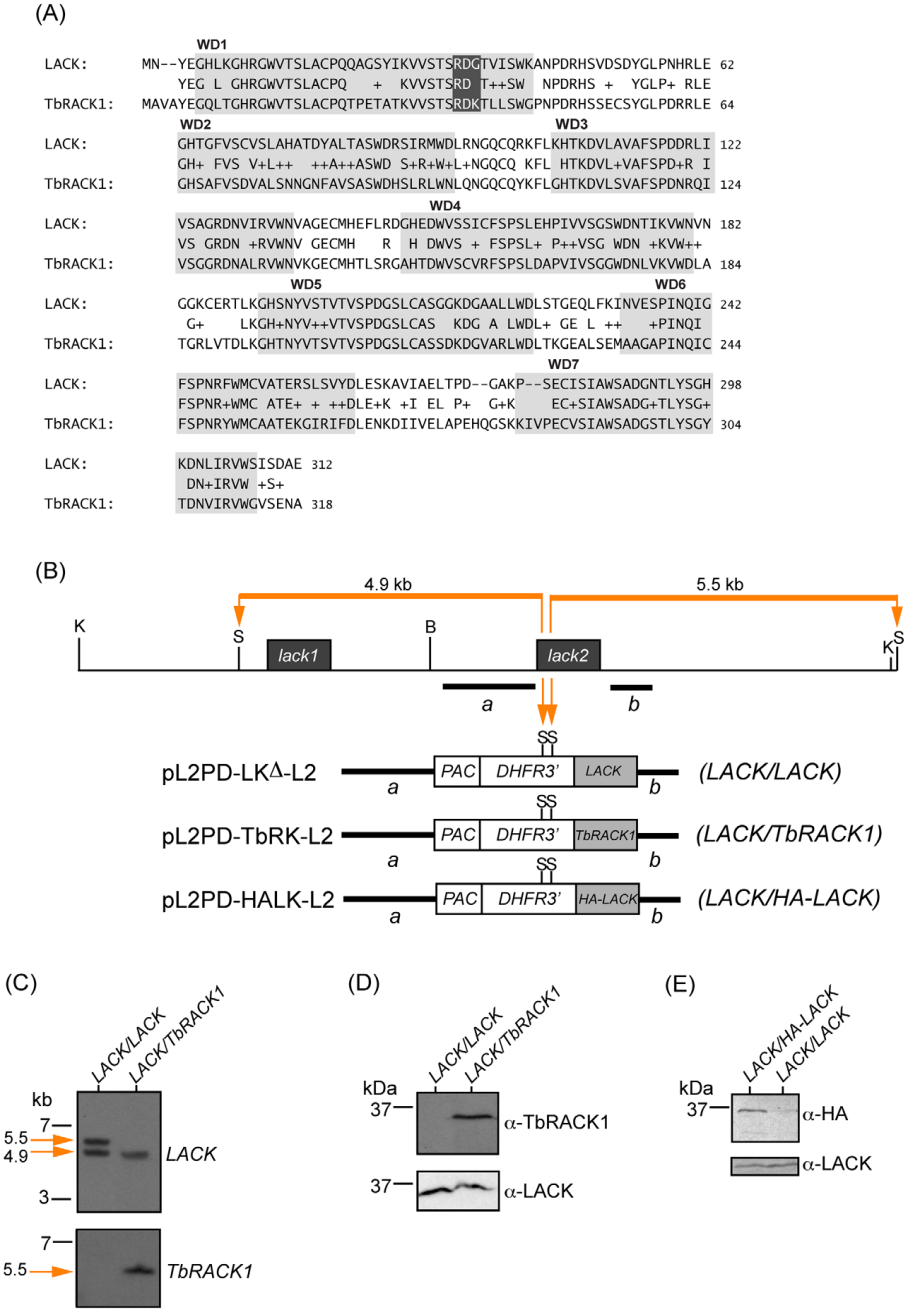
Replacement of the *L. major lack2* gene with *TbRACK1*. (A) Alignment of the protein sequences for *L. major* LACK and TbRACK1, as indicated, was performed using BLAST (bl2seq; blast.ncbi.nlm.nih.gov). Gray boxes denote each WD40 ß-propeller blade domain, numbered as indicated. Identical conserved amino acids between LACK and TbRACK1 are indicated by single letter code. Similar amino acids are indicated by a plus sign. White letters indicate the putative ribosome-binding motif. (B) Physical map of the *L. major lack* genes and targeting constructs used to replace *lack2*. Restriction enzyme sites for *Kpn* I, *Stu* I, and *Bam* HI are denoted as K, S and B, respectively. Targeting fragments *a* and *b* used for the constructs, as described in Materials and Methods, are indicated by heavy black lines. Orange bars and arrows indicate 5.5 kb and 4.9 kb *Stu* I fragments expected following targeting of *lack2* with the indicated constructs. (C) Southern analysis of *L. major* transfectant DNAs. Genomic DNAs from *L. major* transfectants were digested with *Stu* I, size-fractionated and blotted as described in Materials and Methods. The blots were then hybridized with either the *LACK* or *TbRACK1* coding sequences, as indicated at right of panels. (D) Western analysis of *L. major* transfectants. Extracts from 2×10^7^ transfectant parasites for each of the indicated lines were size-fractionated, blotted and probed with either anti-LACK or anti-TbRACK1 antisera, as indicated and described in Materials and Methods.

An alignment of the LACK and TbRACK1 sequence is shown in Figure 3A. Overall, the proteins are 62% identical and 78% similar. As might be expected, sequences within the WD40 repeats are generally more conserved than the intervening sequences. The RDK motif in the first WD40 repeat that is conserved in RACK1 orthologs, but not LACK, is highlighted in the figure. An overview of the gene replacement strategy used to create the *LACK/TbRACK1* strain is shown in Figure 3B. We have used a similar strategy in the past to replace *LACK* alleles. Briefly, the second copy of *LACK* (*lack2*) in the *LACK/LACK* strain was replaced with a copy of *TbRACK1* or *HA*-tagged *LACK* by homologous recombination. Targeting was achieved using *lack2* 5′ and 3′ flanks, along with a marker conferring resistance to puromycin (PAC) to introduce *TbRACK1* or *HA-LACK* into the *lack2* genomic site. Previously, an identical strategy was used with the construct pL2PD-LK^Δ^-L2 to create the *LACK/LACK* strain used in these studies [20]. Southern blots were conducted to confirm successful targeting and strain identity (Figure 3C). As shown in the figure, following transfection of *lack++/−−* with the indicated fragments from pL2PD-LKΔ-L2, pL2PD-TbRK-L2 or pL2PD-HALK-L2 and puromycin selection, *lack2* was replaced by *LACK*, *TbRACK1* or *HA*-tagged *LACK* respectively. Hybridization with *LACK* sequences revealed both *LACK* copies in the *LACK/LACK* (indicated by *LACK*-hybridizing *Stu* I fragments of 5.5 kb and 4.9 kb in Figure 3C, upper panel) and *LACK/HA-LACK* strains (data not shown), and a single copy of *LACK* in the *LACK/TbRACK1* strain (indicated by a single *LACK*-hybridizing fragment of 4.9 kb in Figure 3C, lower panel). Hybridization with *TbRACK1* sequences revealed a single copy of this gene to be present on a 5.5 kb *Stu* I fragment exclusively in the *LACK/TbRACK1* parasites, as expected. Lastly, we confirmed the expression of TbRACK1 and HA-LACK in the *LACK/TbRACK1* and epitope-tagged *LACK/HA-LACK* strains by immunoblot with either anti-LACK, anti-TbRACK1 or anti-HA (Figures 3D and 3E, respectively). As anticipated, TbRACK1 and HA-LACK were expressed in the *LACK/TbRACK1* and *LACK/HA-LACK* strains, respectively, but not the *LACK/LACK* strain. After confirming TbRACK1 expression in the *LACK/TbRACK1* strain, we examined the biological and biochemical properties of this strain relative to *LACK/LACK* parasites. The *LACK/HA-LACK* strain was used for co-immunoprecipitation studies using anti-HA antibodies. We also confirmed that phenotypic differences observed between control and *LACK/-* strains were indeed attributable to LACK deficiency, by introducing a LACK expression plasmid into the *LACK/-* line as previously described [24], see Figure S1.

### Introduction of TbRACK1 rescues the growth defect in LACK/- parasites

**Figure 4:**
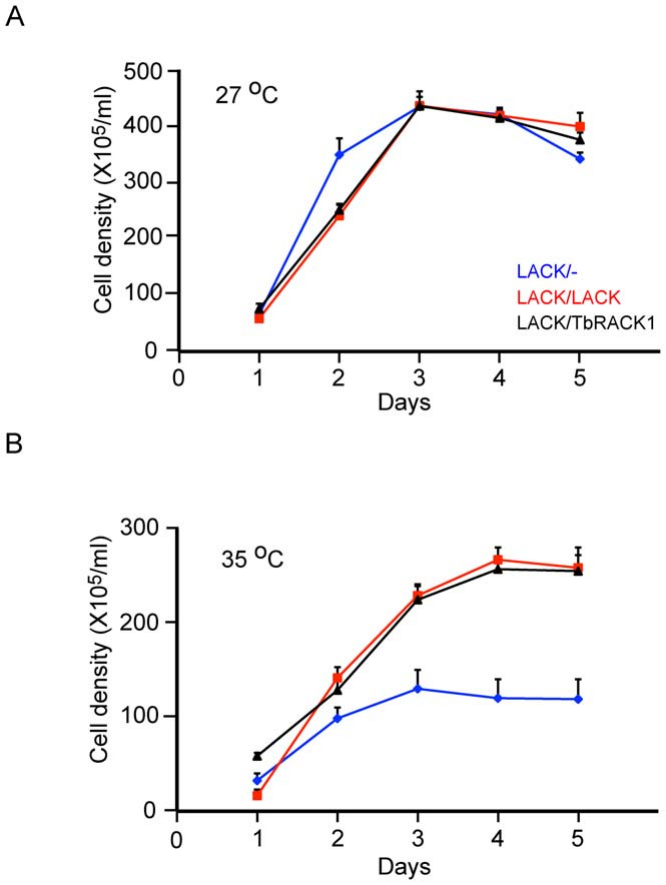
Growth of *LACK/TbRACK1 L. major* at host temperatures. Parasites were inoculated into medium at 5×10^5^/ml and incubated at either 27°C or 35°C as indicated. Cells were counted daily for five days, using a hemocytometer for two independent sets of clones performed at least three times. The data, with standard error bars, are representative of two independent sets of clones repeated three times.

The data in Figure 1 indicate that *LACK/-* parasites have a severe growth defect at the host temperature of 35°C, although their axenic growth at 27°C is less impaired. Therefore, we examined the growth properties of *LACK/TbRACK1* parasites at 27°C and 35°C (Figure 4). As shown in the figure, replacement with *TbRACK1* restores normal growth at 35°C, which is indistinguishable from the growth of *LACK/LACK* parasites.

Having observed *LACK/TbRACK1* parasites to grow with the same kinetics as *LACK/LACK* parasites, we performed biochemical analyses to assess differences in protein synthesis, and LACK (or TbRACK1) associating with the translation apparatus. Because both *LACK/LACK* and *LACK/TbRACK1* parasites grow equivalently, any observed differences can be attributed to differences between LACK and TbRACK1, rather than non-specific effects of cell viability or growth rate.

### TbRACK1 co-sediments with a ribosomal marker in monosome and polysome fractions

**Figure 5:**
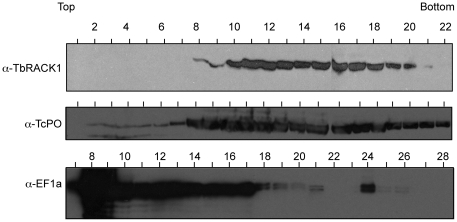
Sucrose gradient polysome analysis of TbRACK1 expressed in *L. major*. *LACK/TbRACK1 L. major* lysates were prepared, fractionated and blotted as described in Fig. 2. Blots were probed with anti-LACK, anti-TcPO and anti- eEF1A antisera as indicated. Fractions are numbered relative to the top and bottom of the sucrose gradient, as indicated.

We examined the distribution of TbRACK1 in monosomes and polysomes using extracts of *LACK/TbRACK1* parasites fractionated on discontinuous sucrose gradients. The distribution of TbRACK1 (Figure 5) was identical to that observed for LACK (Figure 2). TbRACK1 was associated with both monosomes and polysomes, and was absent in non-ribosomal fractions. These results imply that the presence of TbRACK1 in non-ribosomal fractions of fractionated *T. brucei* gradients represent differences in the intracellular milieu between *Leishmania* and *T. brucei* rather than an intrinsic difference between these RACK1 orthologs. Our findings permit us to conjecture that a similar difference may underlie differences in the distribution pattern of RACK1 in *S. cerevisiae* and *S. pombe*.

**Figure 6:**
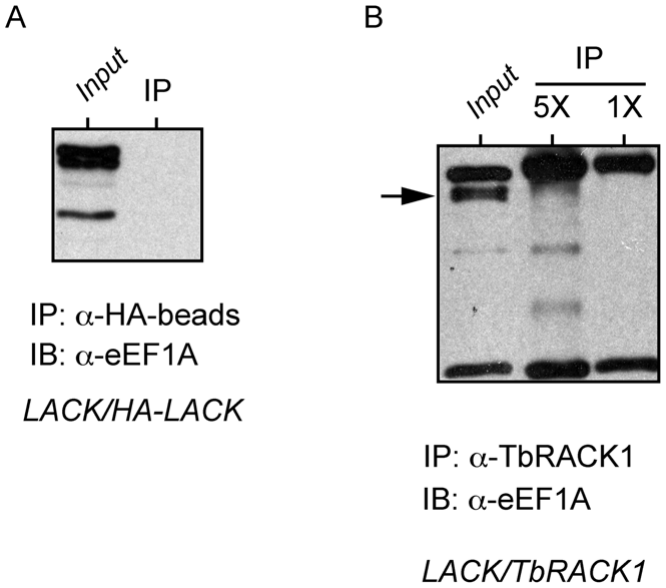
Co-immunoprecipitation analyses of potential interactions between LACK and TbRACK1, and eEF1A. *L. major* lines expressing either an HA-epitope-tagged form of LACK or TbRACK1 were created and analysed as depicted for *LACK/TbRACK1* in Figure 3. Lysates from these two lines were analysed by co-immunoprecipitation under conditions previously described [18], using either anti HA-antibody coupled to agarose beads or anti-TbRACK1 antisera incubated with protein A/G agarose in panels A and B, respectively. Total lysate inputs are indicated. Note that for the total lysate input in Figure 6B, the immunoprecipitating antibody has also been added to the lysate. Arrow indicates *L. major* eEF1A.

Previous studies in *T. brucei* indicate that TbRACK1 interacts with translation elongation factor eEF1A, and indeed co-sediments with eEF1A in the non-ribosomal fractions of *T. brucei* extracts on sucrose gradients [18]. eEF1A from *T. brucei* and *L. major* are 92% identical, and therefore we were surprised to find that in *LACK/TbRACK1 L. major*, TbRACK1 was excluded from non-ribosomal fractions of this gradient. To further elucidate an association between TbRACK1 or LACK1 with eEF1A in *Leishmania* extracts, we performed co-immunoprecipitations using an antibody against eEF1A. Reciprocal co-immunoprecipitations were also performed using antibodies against LACK, HA-epitope and TbRACK1. In repeated attempts, we did not detect an association between LACK, HA-LACK or TbRACK1 and *Leishmania* eEF1A (Figure 6 and data not shown). Because *Leishmania* eEF1A is 92% identical to *T. brucei* eEF1A, our results imply that in *T. brucei*, TbRACK1 interacts with eEF1A through other proteins that must differ between *Leishmania* and *T. brucei*. This finding correlates with the previously published observation that recombinant TbRACK1 interacts with eEF1A in extracts of *T. brucei*, or in extracts partially purified using a calmodulin column, but not when *E. coli* –expressed recombinant forms of both proteins obtained were used [18]. Our results suggest that this interaction may occur through a *T. brucei*-specific intermediate that is present in *T. brucei* extracts or co-purifies with eEF1A on a calmodulin column, and therefore is not observed when both proteins were expressed in a recombinant form.

Irrespective of a functional interaction with eEF1A, we were curious whether LACK's co-sedimentation with monosomes and polysomes correlated with a function in translation, similar to TbRACK1 and other RACK1 orthologs. Because *LACK/-* parasites are attenuated in virulence and growth at 35°C, we hypothesized this difference to arise from defects in translation that can be overcome by additional LACK protein. A corollary suggested by this hypothesis is that exposure to translation inhibitors may further discriminate between *LACK/-* and *LACK/LACK* parasites. This was tested by exposing parasites to inhibitors of translation initiation and elongation.

### LACK/- parasites are more susceptible to translation inhibitors than LACK/LACK and LACK/TbRACK1 parasites

Because recent evidence indicates roles for LACK in translation elongation [18] and initiation [11], [16], we tested the effect of the elongation inhibitor, anisomycin [25], [26], and the initiation inhibitor, hippuristanol [27], on the *Leishmania* strains used in this report. By occupying the A-site, anisomycin inhibits translation elongation via interfering with the peptidyl transferase activity of the 80S ribosome. Hippuristanol binds the eukaryotic translation initiation factor 4 alpha (eIF4A) and inhibits its essential function as an RNA helicase to block initiation.

**Figure 7:**
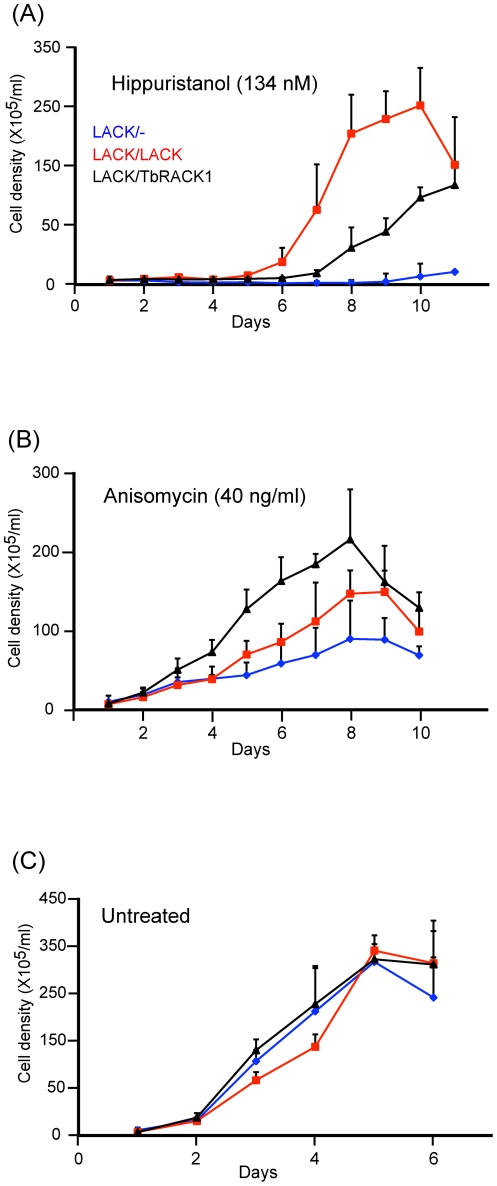
Effects of translation inhibitors on parasite growth. The indicated parasite lines were seeded in medium 199/10% FBS at 5×10^5^/ml and incubated at 27°C in the presence of the indicated concentrations of the translation inhibitors hippuristanol (A) and anisomycin (B), or no inhibitor (C). Cell densities were determined daily for seven days by enumeration, using a hemocytometer for two independent sets of clones performed at least twice. The data were averaged and plotted with standard error bars as indicated.

As shown in Figure 7A, growth of *LACK/-* parasites was severely affected by exposure to hippuristanol. This growth defect was somewhat ameliorated in *LACK/TbRACK1* parasites, and to a significantly greater extent in *LACK/LACK* parasites. This result indicates that either *LACK/LACK* parasites are more resistant to translation inhibitors, or that the functions of LACK and TbRACK1 differ in translation initiation.

Next, we tested the effect of anisomycin on the growth of *LACK/-*, *LACK/TbRACK1* and *LACK/LACK* parasites. Once again, as shown in Figure 7B, *LACK/-* parasites were more susceptible to anisomycin than the other strains. However, *LACK/TbRACK1* parasites were more resistant to anisomycin than *LACK/LACK* parasites. Therefore it is unlikely that the differential effect of hippuristanol on *LACK/LACK* and *LACK/TbRACK1* parasites represents a general property of the former. Rather, it is simpler to interpret that the function of LACK in translation subtly differs from the function of TbRACK1. Both proteins function in translation with differential emphases on initiation and elongation.

### TbRACK1 restores virulence to LACK/- parasites

**Figure 8:**
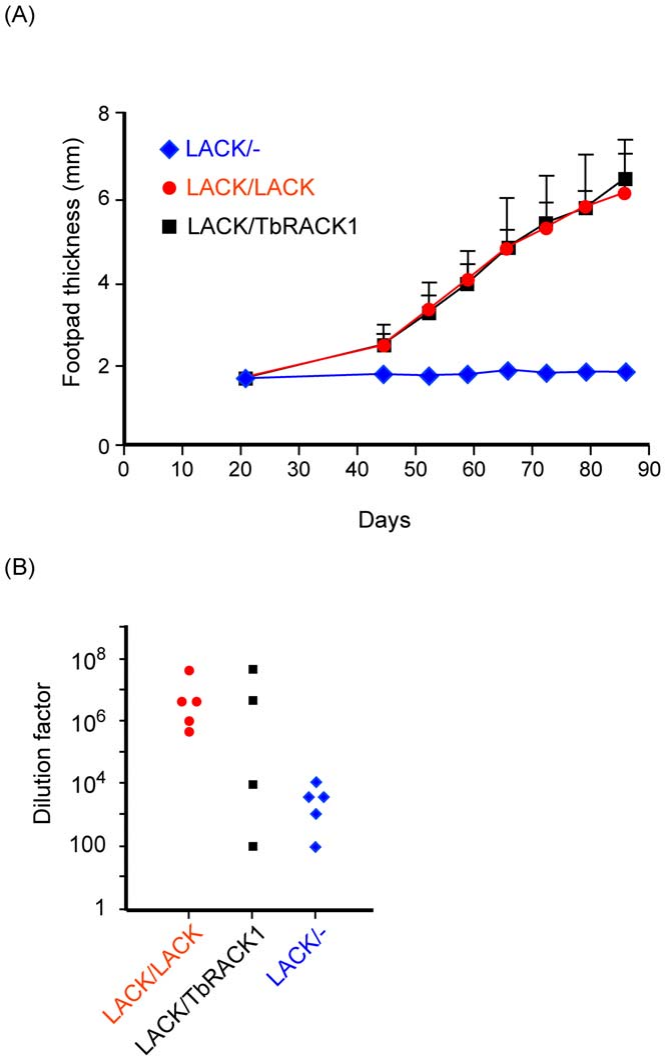
Disease development and parasite burden following infection with *LACK/TbRACK1 L. major*. (A) BALB/c mice were inoculated in the left hind footpad with 4×10^6^ of each of the indicated *L. major* strains. Disease progression in the hind footpad was monitored with vernier-reading calipers (Mitutoyo, Japan) as indicated. (B) For parasite burden determinations, parasites were isolated from popliteal lymph nodes at the experimental endpoints for each parasite line, dispersed in medium 199 and decimally diluted. Parasite burdens for each strain, as indicated in the Figure, were determined by identifying the highest dilution from which viable parasites were recovered.

The preceding data, demonstrate that although introduction of *TbRACK1* into *LACK*-deficient parasites can restore viability and increased resistance to translation inhibitors, there are differences between LACK and TbRACK1. We wondered whether these differences would impinge on parasite virulence *in vivo*. Therefore, we compared the *LACK/LACK* and *LACK/TbRACK1* strains for their capacity to induce disease in the susceptible BALB/c mouse strain. Hind footpads of BALB/c mice were injected sub-cutaneously with parasites as described in the methods, after which the development of footpad lesions was monitored (Figure 8). Footpad lesions developed comparably in *LACK/LACK* and *LACK/TbRACK1* infected mice (Figure 8A), in contrast to *LACK/-* infections that resulted in negligible disease as reported previously [20].

Similarly, analyses of parasite burdens from suspensions of popliteal lymph nodes that drain the infected footpad indicated that comparable levels of parasitemia were attained in mice infected with either *LACK/LACK* or *LACK/TbRACK1* parasites (Figure 8B).

Therefore, although there are biochemical differences between LACK and TbRACK1, they are not critical to the development of disease in this animal model.

## Discussion

RACK1 proteins are conserved in all eukaryotes and have multiple physiological roles as adaptor proteins, regulating cellular events including signaling and translation. Although ostensibly, RACK1 proteins show a remarkable degree of overall sequence conservation amongst eukaryotes, recent studies have uncovered functionally important motifs that are not universally conserved amongst RACK1s [21],[28], [29]. For example, RACK1, a substrate and regulator of mammalian Src kinase is phosphorylated on Y228 and Y246 by Src [21], [22], whereas *L. major* LACK has phenylalanine residues (F230 and F248) at the equivalent positions. Such observations indicate that throughout eukaryote evolution, RACK1 proteins have diverged in structurally subtle yet functionally important ways.

Our interest in the constitutively expressed *Leishmania RACK1* ortholog, *LACK* [30], was piqued by the observation that diploid *Leishmania* and other trypanosomatids have four identical copies of this gene. In contrast, other eukaryotes ranging from mammals to yeast have two copies of *RACK1* per diploid genome [31]. Indeed, *RACK1* can be deleted completely from budding and fission yeast without impacting cell survival and proliferation [7], [10]. It is also likely that RACK1 is essential for trypanosomatid survival and proliferation. Depletion of RACK1 by RNA interference in *T. brucei* results in a dramatic defect on cytokinesis and disruption of the cell cycle [19]. While it is not possible to deplete proteins by RNA interference in *L. major*, our gene knockout studies indicate that survival of *L. major* requires at least one copy of *LACK* [20]. Further, strains of *L. major* with a single copy of *LACK* are considerably impaired in their survival at mammalian host temperatures, and severely impaired in pathogenesis. Parallel to observations in *T. brucei*, our findings indicate that *Leishmania* strains with a single copy of *LACK* are impaired in progression through the G2/M phases of the cell cycle, particularly at mammalian body temperature.

In light of studies highlighting a role for RACK1 proteins in regulating eukaryote translation [7], [8], [13], and the emerging dogma that trypanosomatid gene expression relies heavily upon translation regulation [32], [33], we sought to further elucidate the important role of LACK in *L. major* with respect to parasite viability, protein synthesis and virulence.

RACK1 proteins have been shown directly, or indirectly, to interact with ribosomes to modulate translation [13], [18]. This interaction requires a conserved RDK motif present within the first WD40 domain of these RACK1 proteins. This motif is not conserved in the RACK1 orthologs of *Leishmania* or *T. cruzi*; curiously, while biochemical and structural studies indicate that RACK1 directly associates with *S. cerevisiae* ribosomes [12], [13], other studies indicate absence of a direct RACK1 association with *T. cruzi* ribosomes [34]. We interpret this difference to indicate that a single positively charged residue with the RDK motif is insufficient for direct ribosome association. In contrast, co-sedimentation analysis indicates that *T. cruzi* RACK1 co-sediments with *T. cruzi* ribosomes [18], suggesting an indirect association rather than the direct association observed in *S. cerevisiae*. Taken together, these data suggest that two positively charged amino acids in the putative ribosomal binding domain are not an absolute requirement for the association of RACK1s with trypanosomatid ribosomes, or that the association between RACK1 proteins and ribosomes in trypanosomatids is indirect. We interpret our findings to indicate that RACK1 proteins from different species may associate with the translation apparatus in different ways that are likely dependent on the intracellular milieu. For example, in *T. brucei*, RACK1 interacts with eEF1A [18], however our concerted efforts indicate this interaction may not occur in *L. major*. Nevertheless, *T. brucei* RACK1 can complement *LACK/- L. major* strains to restore their growth properties and virulence. Differences between the RACK1 proteins are also indicated by a differential susceptibility to various translation inhibitors by *LACK/LACK* and *LACK/TbRACK1* strains of *L. major*.

Both LACK and heterologously-expressed TbRACK1 co-sediment in monosome and polysome sucrose gradient fractions from *L. major*, but are absent from non-ribosomal fractions. Such profiles are comparable to those observed for RACK1 in humans, mice, *Drosophila*, *C. elegans* and *S. cerevisiae* [10]. In contrast, TbRACK1 is detected in *T. brucei* ribosome-containing, and non-ribosomal fractions, similar to findings with *S. pombe* RACK1 [7], [35]. Although the functional consequences of such species-specific differences in cellular compartmentalization of RACK1 proteins are unclear, our findings may explain why eEF1A, localized predominantly in low molecular weight sucrose gradient fractions, was not detected in *L. major* complexes immunoprecipitated with anti-HA or anti-TbRACK1 antisera. The presence of a minor population of eEF1A in specific polysome fractions is a novel observation and may represent association of eEF1A with non-ribosomal high-order protein complexes [36].

Consistent with roles for RACK1 orthologs in *Leishmania* protein synthesis, *LACK/LACK* and *LACK/TbRACK1* lines were more resistant to translation inhibitors than *LACK/- L. major*. Interestingly, *LACK/LACK* and *LACK/TbRACK1* lines can be distinguished by their resistance to the translation inhibitors hippuristanol and anisomycin. Hippuristanol interferes with translation initiation by inhibiting initiation factor eIF4A, a DEAD-box family RNA helicase that unwinds 5′ secondary structures in mRNAs to promote translation initiation [27]. Conversely, anisomycin inhibits peptidyl transferase activity in translation elongation [25], [26]. Previous studies indicate that RACK1 may modulate eIF4A [11]; it is reported that phosphorylation of eIF4A may negatively regulate translation [37] [38]; pertinently, *S. cerevisiae* RACK1 decreases phosphorylation of eIF4A [11]. Our findings indicate that *L. major* strains with a single copy of *LACK*, either *LACK/-* or *LACK/TbRACK1*, display greater sensitivity to the eIF4A inhibitor hippuristanol. These findings imply that abundant LACK levels positively impact the function of eIF4A. Given the well-established roles for RACK1 proteins in regulating protein phosphorylation, one speculative scenario that may account for our findings is that LACK may augment *Leishmania* protein synthesis by down-modulating eIF4A phosphorylation. Further, it appears to do this more efficiently than TbRACK1. Our future investigations will be directed toward understanding the mechanism by which LACK impacts eIF4A function.

Although *LACK/TbRACK1* and *LACK/LACK* strains are distinguished by their sensitivity to translation inhibitors, both strains displayed equivalent virulence in mice. Both strains replicated equivalently at mammalian host temperatures and were recovered comparably from infected mouse footpad lesions. Therefore, although these proteins likely have different biochemical properties, each of them can restore growth and virulence to *LACK/-* strains. Our future studies will explore functions of these proteins necessary for axenic growth, and functions necessary for virulence.

In conclusion, our data are consistent with a model where LACK participates in protein synthesis similar to the function proposed for TbRACK1 in *T. brucei* [18]. The effect of LACK and TbRACK1 on cell cycle progression and cytokinesis raises the possibility that RACK1 proteins may selectively impact translation of specific mRNAs, as proposed previously [17]. Because of the multi-functional nature of RACK1 proteins, however, it is also possible that the role of these proteins in the *Leishmania* cell cycle could involve processes additional to translation. Further, given the recent approval of the translation inhibitor, paromomycin as a treatment against leishmaniasis [39] the differential sensitivities of *L. major* lines, expressing distinct RACK1 orthologs, to the specific translation inhibitors used in our studies, suggest RACK1 proteins as potential species-specific targets for parasite-selective therapies.

## Methods

### Parasite culture

All parasite lines used were derived from *L. major* strain WHOM/IR/-/173 and cultured at either 27°C or 35°C in medium 199,10% heat-inactivated FBS as previously described [20].

### Microscopy

Parasites from 4-day old cultures incubated at either 27°C or 35°C were washed twice in PBS, fixed in 4% formaldehyde/PBS, and applied to poly-lysine-coated glass slides using a Cytospin instrument (Shandon/Thermo Fisher Scientific) in accordance with manufacturers' instructions. The slides were then washed in PBS/0.5% Triton-X100 and mounted in ProLong Antifade with DAPI (Invitrogen). Differential interference contrast (DIC) and DAPI-fluorescent images of the parasites were then obtained using a Leica DM RA2 epifluorescence microscope (Leica Microsystems) equipped with the appropriate filters, excitation sources and motorized z-stage controlled by Slide Book software (Intelligent Imaging Innovations).

### Image analysis

DIC microscopic images were analysed to quantify promastigote flagellar lengths using open source ImageJ software, available free of charge at http://rsb.info.nih.gov/ij/.

### Cell cycle analysis

Parasites cultured for four days at 27°C or 35°C were fixed at −20°C with 90% ice cold methanol for 1 hr, then stained with propidium iodide as previously described [40] and analysed following doublet exclusion, using a FACSCalibur flow cytometer and Cellquest software (BD Scientific).

### Polysome analyses

Sucrose gradient fractionation analyses of *Leishmania* polysomes were performed as described previously [41]. Briefly, 0.5–1×10^9^ cells were pelleted, resuspended in 5 ml medium 199 containing 100 µg/ml cycloheximide for 5 min at room temperature. The cells were then washed twice in 50 ml ice-cold PBS containing 100 µg/ml cycloheximide. The cells were then resuspended in 50 ml lysis buffer (15 mM Tris-HCl (pH 7.4), 0.3 M KCl, 5 mM MgCl_2_) containing 0.5 mM DTT, 100 µg/ml cycloheximide, 1 mg/ml heparin, 1× Complete Mini protease inhibitor cocktail (Roche), 120 units of RNAsin (Promega Inc.) and incubated on ice for 10 min. The cells were then lysed by addition of 25 µl of 20% Triton-X 100. Lysates were then centrifuged at 12,000× g for 15 min at 4°C. Lysate supernatants were then layered onto 11 ml 10–50% sucrose step gradients made up in polysome buffer (20 mM Tris-HCl (pH 8.0), 140 mM KCl, 5 mM MgCl_2_) and centrifuged for 160 min at 35,000 rpm in a SW-40 rotor (Beckman Instruments). 0.5 ml fractions were then collected from the top of the gradient using a Foxy Jr. fraction collector (Teledyne Isco, Inc.). Proteins were isolated from each fraction by methanol/chloroform precipitation as previously described [18] and submitted for Western analysis.

### Immunoblotting

Lysates from 2×10^7^ parasites per lane were prepared using 2× Laemmli buffer and run on 12% SDS-PAGE gels and blotted onto PVDF membranes (ImmunBlot, BioRad) according to the manufacturer's instructions. After overnight blocking in 5% milk powder [20] in TBS with 0.05% Tween 20 (TBS/T) at 4°C, blots were incubated with 1∶1,000 rabbit anti–LACK antiserum, 1∶2000 rabbit anti-TbRACK1 (kindly provided by Dr. Larry Ruben, Southern Methodist University, Dallas), 1∶2500 rabbit anti-TcPO antiserum (a generous gift from Drs. Ajay Bhatia and Steven Reed, Infectious Disease Research Institute, Seattle) and 1∶3000 mouse anti-EF1A (Upstate Biotechnology, Lake Placid) for 1 h at 37°C. After washing in TBS/T, blots were incubated with 1∶3000 goat anti–rabbit-Ig conjugated with horseradish peroxidase for 30 min. The blots were washed and developed using ECL chemiluminescence reagent (GE Healthcare) according to the manufacturer's instructions.

### Construction of the TbRACK1 and HA-LACK targeting plasmids

The *TbRACK1* coding sequence was obtained by PCR amplification from genomic DNA isolated from *T. brucei* strain 427 procyclic stages, kindly provided by Dr. Zachary Mackey, University of California, San Francisco.

The *TbRACK1*-targeting construct, pL2PD-TbRK-L2, was created as described previously for pL2PD-LK^Δ^-L2 [20], except that the *TbRACK1* coding sequence was inserted between the puromycin selection cassette and the 0.6 kb *lack2* 3′ flank in pL2PDL2.The *HA-LACK*-targeting plasmid was created in similar fashion to pL2PD-TbRK-L2, except the *HA-LACK* coding sequence was obtained by PCR amplification of the *LACK* gene, using a 5′ primer encoding methionine, followed by three HA epitope sequences, separated by serine-glycine dipeptide linkers, followed by *LACK* codons two, to nine.

### Replacement of lack2 with TbRACK1 and HA-LACK

The downstream *lack2* gene from *lack++/−− L. major* was replaced with a cassette containing either the *TbRACK1* or the *HA-LACK* open reading frame, by transfecting the *lack++/−−* line [20] with the linear insert purified from pL2PD-TbRK-L2 or pL2PD-HALK-L2 (Fig. 4B), using methods previously described [20]. Generation of *LACK/LACK* lines, previously termed *lack++* ^Δ^ */−−*, has been described previously [20].

### Southern blot analysis

Genomic DNAs isolated from control *L. major* transfectant clones previously described [20] and *LACK/TbRACK1 L. major* were digested with *Stu* I and size fractionated by agarose gel electrophoresis. The nucleic acids were blotted onto Hybond N+ nylon membranes (GE Healthcare) and hybridized using Rapid-Hyb (GE Healthcare) with DNA fragments *g* or *h* corresponding to the *lack1* coding region and the 3′-most region of the *lack* allele, respectively (Fig. 4 B), according to the manufacturer's instructions (Phototope, NEB). Blots were washed at 65°C with 2X SSC-0.2X SSC/0.5% SDS, as described earlier [20]. Hybridizing fragments were visualized by chemiluminescent detection according to manufacturer's protocols (Phototope, New England biolabs). A similar strategy was used to create an *L. major* line where the downstream *lack2* gene was replaced with a sequence encoding an *HA*-epitope-tagged version of *LACK* (data not shown).

### Co-immunoprecipitation assays

HA-tagged LACK or TbRACK1 was immunoprecipitated from *LACK/HA-LACK* and *LACK/TbRACK1* parasite lysates, respectively, using procedures and conditions described previously [18]. HA-LACK was immunoprecipitated under these conditions using ProFound anti-HA agarose beads (ThermoScientific/Pierce), in accordance with manufacturers' protocols. TbRACK1 was immunoprecipitated using anti-TbRACK1 anti-serum, kindly provided by L. Ruben, Southern Methodist University, Dallas, with protein A/G PLUS-Agarose (Santa Cruz Biotechnology) in accordance with manufacturer's directions.

### Translation inhibitors and growth assays

For growth assays, parasites were diluted to 5×10^5^/ml in fresh medium and incubated at 27°C or 35°C. Parasites were fixed in 0.4% formaldehyde/PBS then enumerated daily using a hemocytometer. Inhibitory concentrations of translation inhibitors anisomycin (40 ng/ml) (Sigma-Aldrich, St. Louis, MO), and hippuristanol (134 nM) (kindly provided by Dr. Jerry Pelletier, McGill University, Montreal) that were sub-lethal to *LACK/LACK L. major* were determined empirically such that *LACK/LACK* parasite cell densities peaked at approximately 1–2.5×10^7^/ml.

### Parasite infections

Cohorts of five female BALB/c mice (The Jackson Laboratory, ME) were inoculated subcutaneously in the left hind footpad with 4×10^6^ stationary phase promastigotes, prepared as previously described [20]. Lesions were measured weekly using a Vernier-reading caliper. At the experiment termination point, popliteal lymph nodes were collected from infected mice, resuspended in M199/10% FCS, and diluted decimally into duplicate microtiter wells. After 7 d of culture at 27°C, parasites were quantified by microscopic evaluation of the highest dilutions containing viable organisms.

### Animal ethics statement

All animal infection experiments were performed in strict accordance with the recommendations in the Guide for the Care and Use of Laboratory Animals of the National Institutes of Health. The infection protocol was approved by the Louisiana State University Health Sciences Center Institutional Animal Care and Use Committee (IACUC), IACUC protocol number 2584. All infections were performed under isoflurane anesthesia, and all efforts were made to minimize discomfort.

## Supporting Information

Figure S1 **Effects of elevated temperature and translation inhibitors on** ***LACK/- L. major*** **complemented with a** ***LACK*** **expression plasmid.** (A) Western analysis of *LACK/-/*pXGLACK transfectants. Extracts from 2×10^7^ *LACK/- L. major* and *LACK/- L. major*, complemented by expression of epitope-tagged LACK from the expression plasmid pXGLACK, as previously described [24], were size-fractionated, blotted and probed with anti-LACK antisera as described in Materials and Methods. Upper arrowed band denotes the epitope-tagged LACK protein; middle band indicates endogenous LACK, faint lower band represents an unknown cross-reacting protein. (B) Growth of *LACK/-/pXGLACK* transfectants at host temperatures. Cell densities of the indicated parasite lines were determined daily for seven days, by enumeration as described in the legend to Figure 4. The *LACK/LACK* line is included as a positive control. (C) Effect of hippuristanol on *LACK/-/*pXGLACK transfectants. Cell densities of the indicated parasite lines incubated in medium containing 134 nM hippuristanol were determined by enumeration daily for seven days, as described in the legend to Figure 7. The *LACK/LACK* line is included as a positive control.
(TIF)
