# Supplementary figures and images for: Trypanosomatid RACK1 Orthologs Show Functional Differences Associated with Translation Despite Similar Roles in Leishmania Pathogenesis
Source: PLoS One. 2011 Jun 3;6(6):e20710. doi: 10.1371/journal.pone.0020710 (PMC3108995; doi:10.1371/journal.pone.0020710)

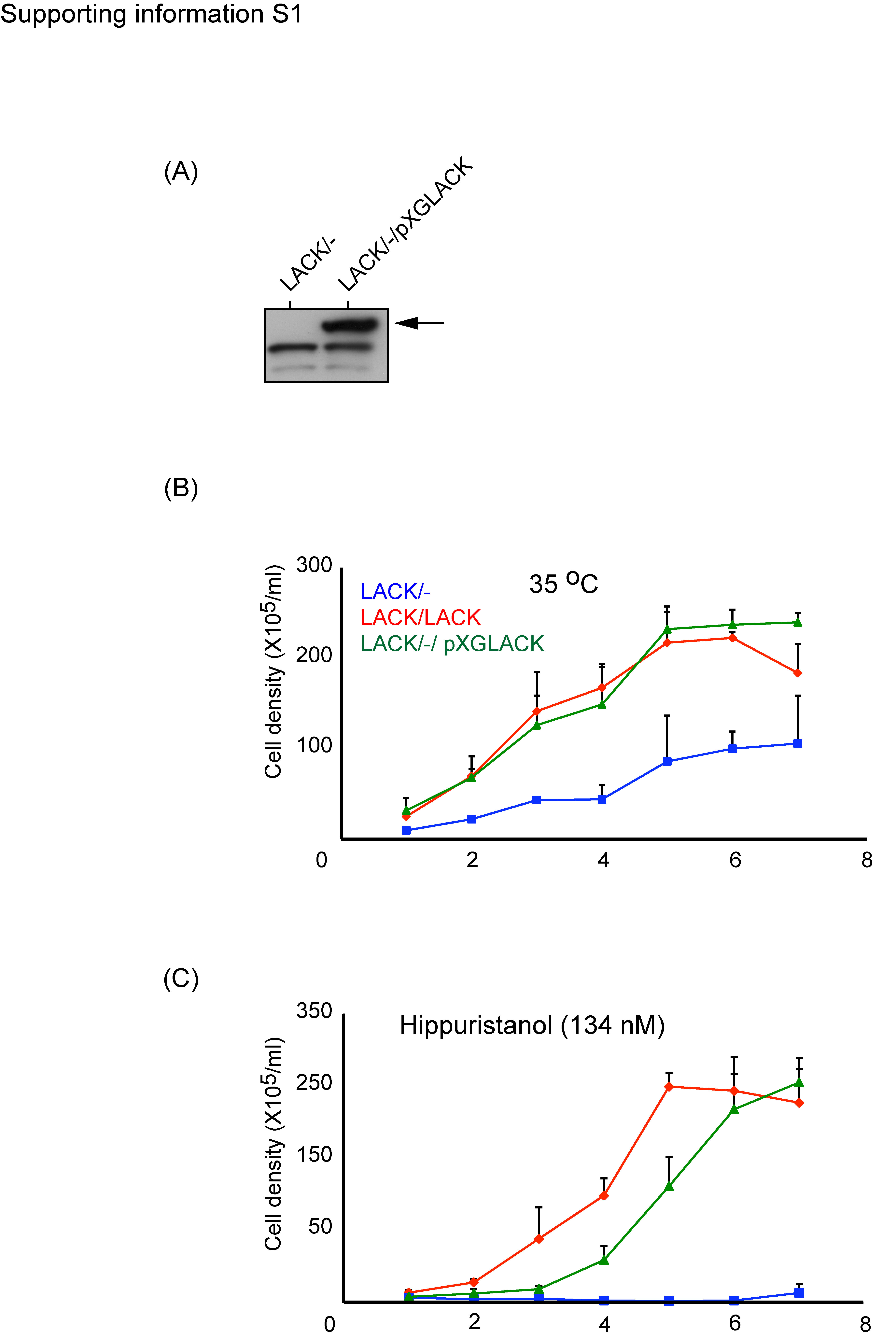

Supplement: Figure S1 — Effects of elevated temperature and translation inhibitors on LACK/- L. major complemented with a LACK expression plasmid. (A) Western analysis of LACK/-/pXGLACK transfectants. Extracts from 2×10^7^ LACK/- L. major and LACK/- L. major, complemented by expression of epitope-tagged LACK from the expression plasmid pXGLACK, as previously described [24], were size-fractionated, blotted and probed with anti-LACK antisera as described in Materials and Methods. Upper arrowed band denotes the epitope-tagged LACK protein; middle band indicates endogenous LACK, faint lower band represents an unknown cross-reacting protein. (B) Growth of LACK/-/pXGLACK transfectants at host temperatures. Cell densities of the indicated parasite lines were determined daily for seven days, by enumeration as described in the legend to Figure 4. The LACK/LACK line is included as a positive control. (C) Effect of hippuristanol on LACK/-/pXGLACK transfectants. Cell densities of the indicated parasite lines incubated in medium containing 134 nM hippuristanol were determined by enumeration daily for seven days, as described in the legend to Figure 7. The LACK/LACK line is included as a positive control. (TIF) [file pone.0020710.s001.tif]
